# Overexpression of a Water-Forming NADH Oxidase Improves the Metabolism and Stress Tolerance of *Saccharomyces cerevisiae* in Aerobic Fermentation

**DOI:** 10.3389/fmicb.2016.01427

**Published:** 2016-09-13

**Authors:** Xinchi Shi, Yanan Zou, Yong Chen, Cheng Zheng, Hanjie Ying

**Affiliations:** ^1^National Engineering Research Center for Biotechnology, College of Biotechnology and Pharmaceutical Engineering, Nanjing Tech UniversityNanjing, China; ^2^State Key Laboratory of Materials–Oriented Chemical Engineering, College of Biotechnology and Pharmaceutical Engineering, Nanjing Tech UniversityNanjing, China; ^3^Jiangsu National Synergistic Innovation Center for Advanced MaterialsNanjing, China

**Keywords:** *Saccharomyces cerevisiae*, NADH oxidase, stress response, transcriptome analysis, thiamine synthesis, global transcriptional factor HAP4, apoptosis, osmotolerance

## Abstract

Redox homeostasis is fundamental to the maintenance of metabolism. Redox imbalance can cause oxidative stress, which affects metabolism and growth. Water-forming NADH oxidase regulates the redox balance by oxidizing cytosolic NADH to NAD^+^, which relieves cytosolic NADH accumulation through rapid glucose consumption in *Saccharomyces cerevisiae*, thus decreasing the production of the by product glycerol in industrial ethanol production. Here, we studied the effects of overexpression of a water-forming NADH oxidase from *Lactococcus lactis* on the stress response of *S. cerevisiae* in aerobic batch fermentation, and we constructed an interaction network of transcriptional regulation and metabolic networks to study the effects of and mechanisms underlying NADH oxidase regulation. The oxidase-overexpressing strain (NOX) showed increased glucose consumption, growth, and ethanol production, while glycerol production was remarkably lower. Glucose was exhausted by NOX at 26 h, while 18.92 ± 0.94 g/L residual glucose was left in the fermentation broth of the control strain (CON) at this time point. At 29.5 h, the ethanol concentration for NOX peaked at 35.25 ± 1.76 g/L, which was 14.37% higher than that for CON (30.82 ± 1.54 g/L). Gene expression involved in the synthesis of thiamine, which is associated with stress responses in various organisms, was increased in NOX. The transcription factor HAP4 was significantly upregulated in NOX at the late-exponential phase, indicating a diauxic shift in response to starvation. The apoptosis-inducing factor Nuc1 was downregulated while the transcription factor Sok2, which regulates the production of the small signaling molecule ammonia, was upregulated at the late-exponential phase, benefiting young cells on the rim. Reactive oxygen species production was decreased by 10% in NOX, supporting a decrease in apoptosis. The HOG pathway was not activated, although the osmotic stress was truly higher, indicating improved osmotolerance. Thus, the NADH oxidase can regulate the metabolism during aerobic fermentation in *S. cerevisiae*, thereby protecting cells against several stresses. Our findings indicate its suitability for use in industrial processes.

## Introduction

Metabolic engineering has generally focused on the directed improvement of cellular properties through the modification of specific biochemical reactions or the introduction of new ones (Stephanopoulos, 1999). However, researchers are beginning to realize that metabolic networks cannot be simply represented as assemblies of genes and proteins with functional properties plainly emerging from their interconnections (Nielsen, 2003). Instead, the potential relationship between the structure and functionality of metabolites and cofactors in a metabolic network needs to be investigated before one can proceed to modifying an existing or constructing a new network (Jeong et al., 2000; Monk et al., 2013).

Cofactors supply Gibbs free energy, redox equivalents, and functional groups in metabolic networks (Zhao and van der Donk, 2003; Liu and Wang, 2007). They generally are the most tightly connected nodes of a network. The intracellular redox potential is primarily determined by the NADH/NAD^+^ ratio and to a lesser extent by the NADPH/NADP^+^ ratio (Vemuri et al., 2007). In *Saccharomyces cerevisiae*, these cofactors are involved in ≥200 reactions spanning a wide range of cellular functions (Doerks et al., 2002). Because NADH is a highly connected metabolite in the metabolic network (Doerks et al., 2002), any change in the NADH/NAD^+^ ratio leads to widespread metabolic changes (Nielsen, 2003). Overexpression of the water-forming NADH oxidase encoded by the *noxE* gene from *Lactococcus lactis* regulated the intracellular redox balance in *S. cerevisiae* by relieving cytosolic NADH accumulation, resulting in substantially lower glycerol synthesis (Heux et al., 2006; Hou et al., 2009, 2010). Heterologous expression likely induces a wide-ranging response instead of affecting a specific metabolic reaction, with network effects centering on the altered reaction (Holm et al., 2010). Kim et al. (2015) found that NADH oxidase overexpression increases 2,3-butanediol production in Pdc-deficient *S. cerevisiae*. Hou et al. (2014) showed that it decreased xylitol and glycerol production in xylose-metabolizing yeast. Moreover, our previous study demonstrated increased cell growth and ethanol production, and decreased glycerol production in anaerobic conditions upon overexpression of NADH oxidase in *S. cerevisiae* (Shi et al., 2016).

Thiamine is another important metabolic cofactor, which can alleviate redox stress, in *S. cerevisiae* (Alff-Tuomala et al., 2016). The active form of thiamine is ThdP (Hohmann and Meacock, 1998). HET is a precursor of thiamine, and ADT, the conversion of which is catalyzed by Thi4, is the precursor of HET (Faou and Tropschug, 2004), and NAD^+^ is the source of the carbohydrate precursor of ADT (Jurgenson et al., 2006; Chatterjee et al., 2007). Since overexpression of NADH oxidase increases the regeneration of NAD^+^, it might also regulate the synthesis of ADT, thus influencing the synthesis and metabolism of thiamine. This point of view has not been investigated to date.

A study by Hou et al. (2009) revealed increased glucose consumption in an NADH oxidase-overexpressing strain, suggesting that it would confront with starvation stress when glucose was depleted while the normal strain would not. Diauxic shift is a type of starvation stress response that occurs when yeast cells have consumed all glucose fermentatively and resort to oxidative catabolism of the remaining ethanol (Lascaris et al., 2002), indicating that the NADH oxidase-overexpressing strain underwent the diauxic shift earlier than the control strain. Overexpression of the global transcriptional factor HAP4 provokes changes that also occur during the diauxic shift (Blom et al., 2000; Lascaris et al., 2002). To our knowledge, a relationship between NADH oxidase and HAP4 has not been reported as so far.

Xu et al. (2010) found that overexpression of a heterologous water-forming NADH oxidase in *Torulopsis glabrata* resulted in decreased ROS production and protected the yeast cells from ROS-induced damage. In addition, it improved the osmotolerance of the cells (Blom et al., 2000; Xu et al., 2010). As NADH oxidase overexpression in *S. cerevisiae* might improve the osmotolerance of cells under high osmotic stress, lack of induction of the HOG pathway—one of the best-studied classical MAPK pathways—might suppress or delay apoptosis. However, this has not been reported to date.

The construction of a global interaction network, including transcriptional regulation and metabolic networks, to integrate data from transcription profiles and the metabolite levels (Holm et al., 2010), is an efficient method to study the mechanisms underlying some specific regulation (Vemuri et al., 2007; Celton et al., 2012) or changes in growth conditions (Li et al., 2015). To better understand the mechanisms of NADH oxidase regulation, comparative RNA-seq analysis of a *S. cerevisiae* control strain (CON) and NADH oxidase strain (NOX) was conducted, with a focus on the stress response.

## Materials and Methods

### Construction of the Yeast Strains

The strains and plasmids used in this study are listed in **Table 1**. The *noxE* (GenBank Accession No. AM406671) gene encoding the water-forming NADH oxidase from *L. lactis* was PCR-amplified with primers Nox-F, 5′-*CTTGTGGGCCCA*GGATCCATGAAAATCGTAGTTATCG-3′, and Nox-R, 5′-*ACAGGAATTCACCAT*GGATCCTTATTTGGCATTCAAAGCTG-3′. Both primers have a BamHI site (underlined), and the homologous arms of the plasmid are indicated in italics in the primer sequences. PCR products were gel-purified and inserted into the *Bam*HI site of pYX212 by using the ClonExpress^TM^One Step Cloning Kit (Vazyme Biotech, Co., Ltd, Nanjing, China), yielding pYX212-NOX. The plasmid was transformed into the host strain, BY4741, using G418 (400 μg/ml) to select a stably transfected clone, designated NOX (**Table 1**). As a control, CON, the host strain transfected with empty plasmid, was used.

**Table 1:** List of plasmids and strains used in this study.

Plasmid/strain	Genotype	Source
**Plasmid**		
pYX212	2 μ, TPI promoter, AMP^R^	A gift from Prof. Yingjin Yuan (Tianjin University, Tianjin, China)
pYX212-NOX	pYX212 with *nox* from *Lactococcus lactis*	This study
**Strain**		
BY4741	*MATa;ura3;his3;leu2;met15*	A gift from Prof. Yingjin Yuan (Tianjin University, Tianjin, China)
CON	BY4741/pYX212	This study
NOX	BY4741/pYX212-NOX	This study

### Media and Growth Conditions

The strains were maintained on conventional yeast extract peptone dextrose (YPD) agar plates as described previously (Ito et al., 1983). Seed cultures for cultivation were grown at 30°C in 500-ml Erlenmeyer flasks containing 100 ml complex medium A (initial pH 5.2) containing glucose (20 g/L), tryptone (10 g/L; Oxoid), yeast extract (5 g/L; Oxoid), and NaCl (9 g/L) (Barber et al., 2002) on a rotary shaker at 200 rpm. Aerobic fermentations were performed at 32°C in 500-ml Erlenmeyer flasks containing 100 ml complex medium A with 90 g/L glucose (initial pH 5.2) on a rotary shaker at 200 rpm after the addition of 10 ml of OD-standardized seed cultures.

### Metabolite Analyses

The cell density was measured using a BioMate^TM^ 3 spectrophotometer (Thermo Scientific, Waltham, MA, USA) at 600 nm. A small volume (5 ml) of culture was centrifuged at 4,000 × *g* for 10 min. The supernatants were used to determine the concentrations of glucose, ethanol, and glycerol. The glucose and glycerol concentrations were measured by high performance liquid chromatography (Agilent 1100 series; Hewlett–Packard, Palo Alto, CA, USA) with a refractive index detector, using a Benson BP-100 Pb^++^ column (300 mm × 7.8 mm; Benson Polymeric, Inc., Sparks, NV, USA). Ultrapure water was used as the mobile phase at a flow rate of 0.4 ml/min and 80°C. The ethanol concentration was analyzed by gas chromatography using an Agilent HP-INNOWAX column (60 m × 250 μm × 0.5 μm) with a flame ionization detector as described previously (Chen et al., 2014).

### Enzyme Activity

For the determination of NOX activity, *S. cerevisiae* cells were collected by centrifugation at 6,000 × *g* at room temperature. The pellet was resuspended in 50 mM phosphate buffer (pH 7.0). After adding 0.2 g of glass beads (I.D. 0.5 mm, Biospec, Bartlesville, OK, USA) into the cell suspension, the mixture was vortexed vigorously for 1 min and cooled for 4 min three times (Lee et al., 2012). After centrifugation for 3 min at 12,000 × *g* at 4°C, the supernatant crude protein extract was used for the enzyme assay. Total NADH oxidation activity was assayed spectrophotometrically following the method of Vemuri et al. (2007). Protein was quantified by the Bradford method using BSA as a standard.

### Quantification of Intracellular NAD(P)H/NAD(P)

The intracellular concentration of NAD(P)H was determined by the enzyme cycling method of Liu et al. (2013) with modifications. Generally, two tubes of 1-ml sample were taken and cells were collected and dissolved in 0.5 ml 0.1 M NaOH [to assay NAD(P)H] and 0.5 ml 0.1 M HCl [to assay NAD(P)], respectively. The cell lysate was heated at 50°C for 10 min, cooled to 0°C, and centrifuged at 10,000 × *g* for 10 min. The supernatant was used for measurement. One hundred microliters of Tris-HCl (1 M, pH 7.8), 100 μL 4.2 mM MTT, 150 μL 16.6 mM PES, and 100 μL ethanol for the determination of NAD(H) or 100 μL 60 mM glucose 6-phosphate for the determination of NADP(H), were sequentially added to a test tube and kept at 37°C for 5 min in the dark. ddH_2_O and an appropriate amount of supernatant (75 μL in total) were added to 96-well plates. The plates were transferred into a Multi-mode Detection Platform (SpectraMax Paradigm; Molecular Devices, Sunnyvale, CA, USA) and preheated at 37°C for 5 min. Ten microliters of alcohol dehydrogenase [1.5 units/μL, for NAD(H)] or glucose 6-phosphate dehydrogenase [70 units/ml, for NADP(H)] were added to the mixture, and 46 μL of the mixture was added to the 96-well plates to start the reaction. The absorbance at 570 nm was determined. NADH was measured for 10 min with 2-min intervals, and NADPH, NADP, and NAD were measured for 30 min with 5-min intervals.

### Harvest of Cells and RNA Isolation

Cells of the CON and NOX strains were collected during three different growth stages—the beginning (8 h), the middle (22 h), and the end of the exponential phase (29.5 h)—by centrifugation (5,000 × *g*, 5 min, 4°C) and washed twice in PBS (8 g/L NaCl, 0.2 g/L KCl, 1.44 g/L Na_2_HPO_4_, 0.24 g/L KH_2_PO_4_, pH 7.4; Li et al., 2015). The cell pellets were immediately frozen in liquid nitrogen and stored at -80°C. Three samples of the same stage were pooled and homogenized, and total RNA was extracted to prepare staged samples for transcript analyses as described previously (Irie and Kuratani, 2011).

### RNA-seq Analysis

RNA-seq analysis was conducted as described in detail by Li et al. (2015). To improve the reliability of data in each developmental stage, we took 2 G of sequencing data (Cao et al., 2005). Differential expression between two experimental conditions was detected by carrying out a modified *t*-test, with a significance threshold of *P* < 0.05. Only genes displaying a fold-change in the expression level of ≥2 (positive or negative), which corresponds with an absolute log_2_ ratio value ≥1, were considered to be significantly differentially regulated. Because this analysis tests 1000s of hypotheses simultaneously (in determining whether given genes are differentially expressed between two groups), corrections for false positives (type I errors) and false negatives (type II errors) were performed using the false discovery rate (FDR) method (Benjamini and Yekutieli, 2001). An FDR ≤ 0.001 and absolute value of log_2_ ratio ≥ 1 were set as criteria for assessing the significance of differential gene expression. Gene ontology (GO) enrichment analysis and pathway enrichment analysis were performed as in Li et al. (2015). The Illumina sequencing data were deposited into the NCBI database under the accession number SRP072476.

### ROS Analysis

Reactive oxygen species was measured following the method of Xu et al. (2010). A flow cytometer (BD FACS Calibur, Becton, Dickinson and Company, USA) was used to detect the fluorescence intensity of the cell suspension. Cells of CON and NOX strains were collected at 22 h (the mid-exponential phase) and 34 h (the stationary phase). ROS production was expressed as a ratio relative to that in CON cells collected at 22 h.

### Osmolarity Determination

Osmolarity was measured using an automatic cryoscopic osmometer (OSMOMAT 030, GONOTEC GmbH, Berlin, Germany).

### Quantitative Reverse Transcription (qRT)-PCR Analysis

RNA was isolated from cells as described previously (Li et al., 2015). Reverse transcription was performed using the AMV First Strand cDNA Synthesis Kit (Sangon Biotech, Shanghai, China) according to the manufacturer’s instructions. Primer Express software was used for primer design. The analyzed genes and primers used in the analysis are listed in **Table 2**. qRT-PCR assays were performed with the SYBR Green PCR Master Mix (Applied Biosystems, Foster City, CA, USA) on a StepOnePlus Real-Time PCR System according to the manufacturer’s instructions. Three technical replicates were included for each sample. Gene transcript levels were determined according to the 2^-ΔΔCt^ method, using *ACT1* (Yuan and Ching, 2015) as a reference gene for normalizing the gene expression levels. To verify qRT-PCR data, standard deviation values were calculated using Microsoft Excel (Microsoft Corporation, Redmond, WA, USA) and were used to evaluate the repeatability and the reliability of the data.

**Table 2:** Primers used for quantitative real-time PCR and their target genes.

Gene ID	Gene name	Primer sequences
YFL039C	*ACT1* (reference gene)	F: TGGATTCCGGTGATGGTGTT R: TGGCGTGAGGTAGAGAGAAACC
L196579	*noxE*	F: TCAAAAATGGCGCAATCAAG R: CCGCGTAAACATCTGGATCA
YJR158W	*Hxt16*	F: TTTGAGCAACGTGCGTATGG R: CGCCAATGGAACATCCTACA
YDR343C	*HXT6*	F: CGCTGCTATTGCAGAGCAAAC R: CGAGTGGGAGGCTGAGTCA
YGR144W	*THI4*	F: TTTGCCGTTTCTGACGTGATT R: GCGGCGGATAAACCTGAA
YFL058W	*THI5*	F: GGTTACTTCAAGGAGCAAGGTCTAGA R: CAGTGACATCGGAAGGATTGG
YJR156C	*THI11*	F: CAAGAAGGCAACCGACTACGT R: GAGGCTTGAAGTCGATGTATTCTTT
YNL332W	*THI12*	F: GGTTACTTCAAGGAGCAAGGTCTAGA R: CAGTGACATCGGAAGGATTGG
YDL244W	*THI13*	F: CAAGAAGGCAACCGACTACGT R: GAGGCTTGAAGTCGATGTATTCTTT
YKL109W	*Hap4*	F: TGTACCGATCGCCCCAAATA R: TGCCATCGTTTTCGAATTCC
YLR113W	*Hog1*	F: GGGCATTTGGGTTGGTTTG R: TTAATGGCAACTGGCTGAGATG
YKL043W	*Sok2*	F: CGAAACTCCAAACGCATATGC R: AGCCTGAGTTGGCGACGTA
YKL062W	*MSN4*	F: CGGCATTCGACAATAACGTAGA R: GATCCTGAGCCGGAGATGAC
YJL208C	*NUC1*	F: TCGATCCTTCCGGGTTCTT R: CGCGGTTCTGCAGATCATG
YBR240C	*THI2*	F: CGATGTCGTCAGCAGAGGAA R: TCTTCTGGCGGCGATGA
YDL022W	*GPD1*	F: TCAATTTTTGCCCCGTATCTG R: GATAGCTCTGACGTGTGAATCAACA
YHL032C	*GUT1*	F: GCCCCAGCTCGTGAAACA R: GGGCTTTCCGCTGGTTTT

## Results and Discussion

### NADH Oxidase Overexpression Improves Aerobic Glucose Fermentation

**FIGURE 1:**
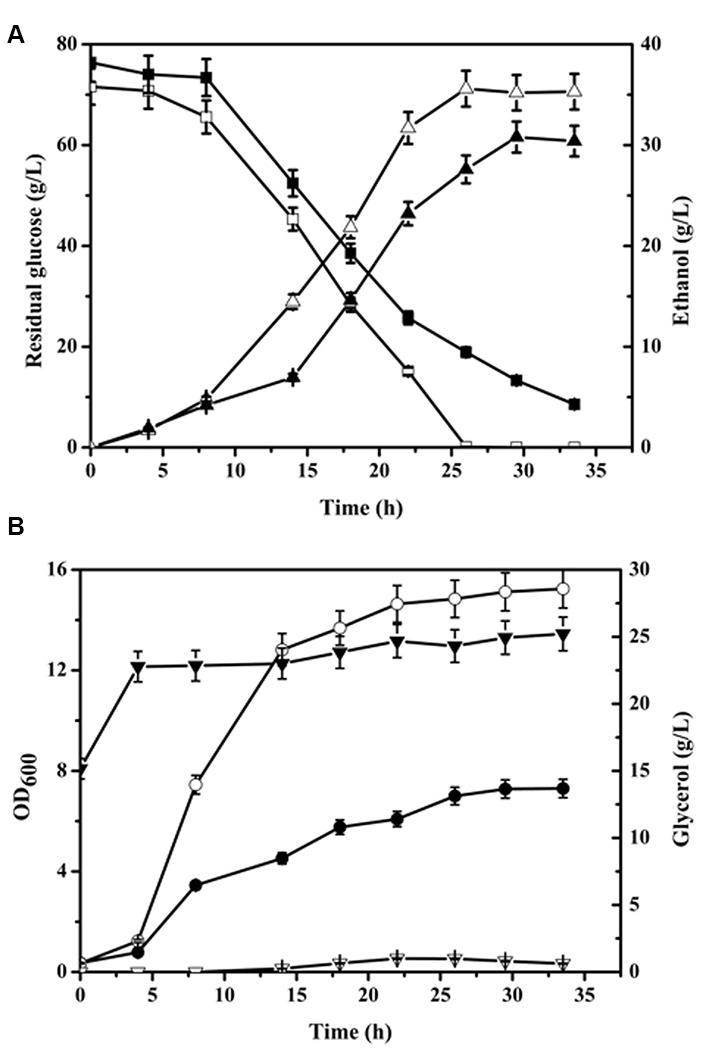
**Time profile of batch fermentation by CON and NOX. (A)** ▲△ represent the time profile of batch ethanol fermentation; ■□, residual glucose; **(B)** ●○, OD_600_; ▼▽, glycerol fermentation. Solid symbols, CON; open symbols, NOX.

Batch culture growth of CON and NOX strains in aerobic condition was compared (**Figure 1**). The glucose consumption and cell growth rate of NOX were higher than those of CON. Glucose was exhausted at 26 h by NOX while 18.92 ± 0.94 g/L residual glucose remained in the CON culture at this time point. Additionally, after 29.5 h of fermentation, the concentration of ethanol produced by NOX peaked at 35.25 ± 1.76 g/L, which was 14.37% higher than that of CON (30.82 ± 1.54 g/L) at this time point. Vemuri et al. (2007) reported an ethanol yield of 0.26 g/g, i.e., 10.4 g/L, in aerobic batch fermentation of *S. cerevisiae* overexpressing a *Streptococcus pneumoniae* NADH oxidase gene. The remarkable increase obtained in our study indicates the potential benefit of our strain for industrial ethanol production. Glycerol production was remarkably lower in NOX, in accordance with previous reports of increased assimilation of NADH in the cytosol by NADH oxidase, leading to a reduction in glycerol production (Heux et al., 2006; Vemuri et al., 2007; Hou et al., 2009). The glycerol concentration of NOX remained under 1 g/L in both the seed cultures and aerobic fermentation processes. CON produced a large amount of glycerol in the seed culture and in the lag phase of fermentation; more than 7 g/L glycerol was produced within 4 h, after which production stably increased. As NOX consumed glucose much faster, it seemed that the NADH oxidase also increased the demand of NADH in aerobic condition since the glycolysis pathway is the main pathway to generate NADH. NADH homeostasis in response to the increased NADH demand was achieved by the regulation of the glycolysis pathway, which was in accordance with a previous report on the NADPH oxidation system (Celton et al., 2012).

In the batch fermentation, the concentrations of the intracellular cofactors were measured at 26, 30, and 34 h. The NADH/NAD^+^ ratio of NOX was higher than that of CON at all three time points, while the NADPH/NADP^+^ ratios were similar between the two strains (**Table 3**). The NADH/NAD^+^ ratios were not consistent with those in previous reports. Vemuri et al. (2007) reported that under either carbon-limited or nitrogen-limited conditions, the NADH/NAD^+^ ratio was 20–50% lower for the NADH oxidase-overexpressing than for the control strain. These inconsistencies may be due to differences in the strains and fermentation conditions used in this and previous studies. First, the parental strains were different, which may have affected the engineered phenotypes. Second, the NADH oxidase genes were from different genomic background; we used the *L. lactis* gene while Vemuri et al. (2007) used a *S. pneumoniae* gene. Third, the fermentation conditions were different; we used complete medium in batch fermentation, while Vemuri et al. (2007) conducted aerobic fermentations under nitrogen-limited and carbon-limited chemostats. Finally, the NADH/NAD^+^ ratio likely is not only determined by the action of the NADH oxidase alone, as the heterologous expression does not affect one specific metabolic reaction.

**Table 3:** The NADH/NAD^+^ ratio and NADPH/NADP^+^ ratio of CON and the NADH oxidase-overexpressing strain NOX.

	NADH/NAD^+^			NADPH/NADP^+^		
	26 h	30 h	34 h	26 h	30 h	34 h
CON	0.29 ± 0.01	0.26 ± 0.01	0.32 ± 0.02	0.77 ± 0.04	0.88 ± 0.03	0.67 ± 0.03
NOX	0.49 ± 0.02	0.49 ± 0.02	0.47 ± 0.02	0.88 ± 0.04	1.00 ± 0.05	0.78 ± 0.04

*Each value is an average of three parallel replicates.*

Additionally, we have measured the NADH/NAD^+^ ratio in the recombinant *S. cerevisiae* strains under various degrees of oxygen supply. The results, which corroborate our findings, have been reported elsewhere (Shi et al., 2016). The NADH/NAD^+^ and NADPH/NADP^+^ ratios in anaerobic and microaerobic conditions showed trends similar to those in aerobic condition; the NADH/NAD^+^ ratios of NOX in the three oxygen supply models were higher than those of CON.

**FIGURE 2:**
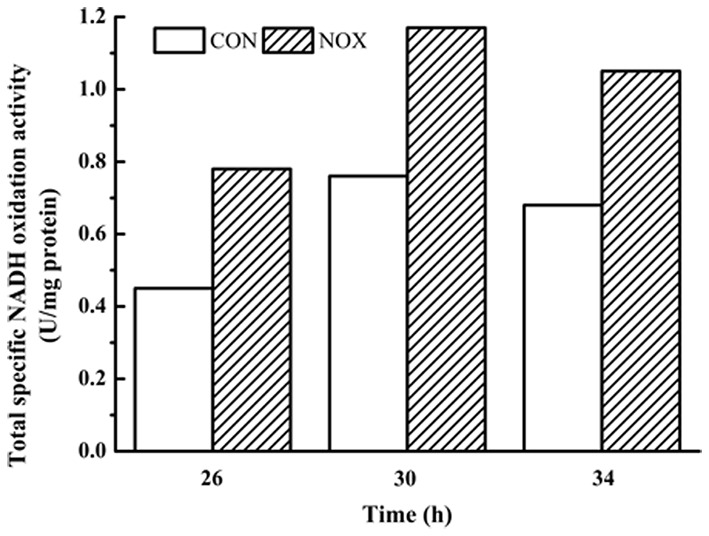
**Total specific NADH oxidation activity in CON and NOX.** In the batch fermentation, the total specific NADH oxidation activities of CON and NOX were measured at 26, 30, and 34 h.

Finally, the NADH oxidation capacity was measured. The assay for determining NADH oxidation is not specific for NADH oxidase and includes native activity that *S. cerevisiae* possesses (e.g., NADH dehydrogenases; Vemuri et al., 2007). As shown in **Figure 2**, NOX consistently exhibited greater NADH oxidation activity than CON at all three time points tested.

### Differential Gene Expression between CON and NOX Cells

**FIGURE 3:**
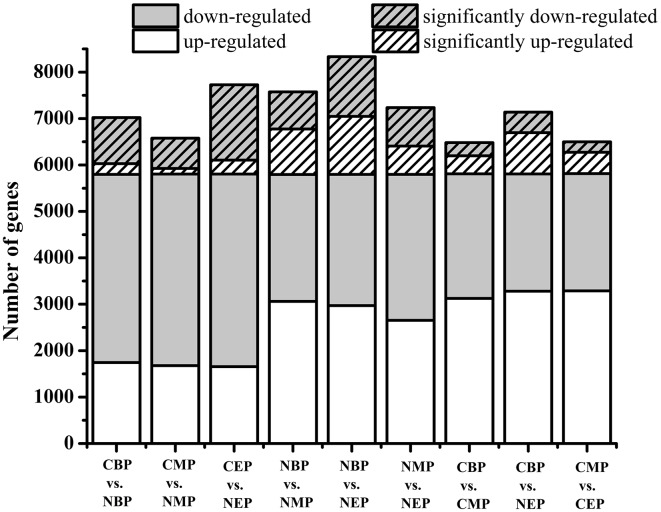
**Differences in gene expression under differing growth conditions.** Hatched bars represent the number of differentially expressed genes whose expression levels were significantly different from those observed in control cells (FDR ≤ 0.001, log_2_ ratio ≥ 1). CBP: CON harvested in the early exponential phase; CMP: CON harvested in the mid-exponential phase; CEP: CON harvested in the late-exponential phase. NBP: NOX harvested in the early exponential phase; NMP: NOX harvested in the mid-exponential phase; NEP: NOX harvested in the late-exponential phase.

mRNA fractions purified from CON and NOX cells at the beginning, middle, and end of the exponential phase were analyzed to determine their respective gene expression profiles and to identify differentially expressed genes. At the early exponential phase, 5798 genes were differentially expressed in NOX vs. CON, of which 992 were significantly downregulated and 230 were significantly upregulated (**Figure 3**). In cells harvested during the mid-exponential phase, 773 genes were significantly differentially expressed in NOX vs. CON, of which 651 were significantly downregulated and 122 were significantly upregulated. At the late-exponential phase, 1621 genes were significantly downregulated and 301 genes were significantly upregulated in NOX vs. CON. Interestingly, in NOX, the number of downregulated genes at the beginning of the exponential phase was 2.32-fold higher than that of upregulated genes, and the ratio was 2.46 in the mid-exponential phase and 2.50 in the late-exponential phase, thus seemingly increasing with longer fermentation. The total number of significantly upregulated genes changed only slightly during the fermentation. However, the number of significantly downregulated genes decreased markedly from the beginning to the middle of the exponential phase and then markedly increased toward the end of this phase. Taken together over all three phases, the numbers of the significantly downregulated genes were over fourfold higher than the number of significantly upregulated genes. As shown in **Figure 3**, gene expression also substantially changed within NOX and CON cells during the fermentation process (columns 4–9). These comparisons indicated that a multitude of genes were differentially regulated during fermentation, as expected, and more dramatic differences were observed in NOX cells than in CON cells.

**FIGURE 4:**
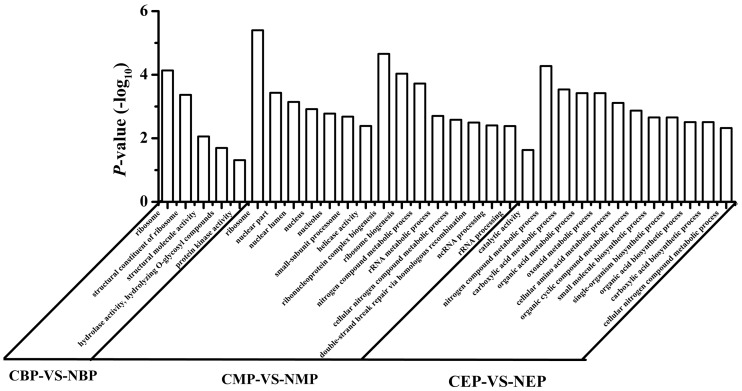
**Gene ontology (GO) category enrichment.** Gene expression levels of CON cells grown for different periods were set as the background level. A corrected *P*-value ≤ 0.05 was used as a threshold. GO terms fulfilling this condition are defined as significantly enriched GO terms for the differentially expressed genes. CBP: CON harvested in the early exponential phase; CMP: CON harvested in the mid-exponential phase; CEP: CON harvested in the late-exponential phase; NBP: NOX harvested in the early exponential phase; NMP: NOX harvested in the mid-exponential phase; NEP: NOX harvested in the late-exponential phase.

The differentially expressed genes were mapped to GO ontologies of cellular components, molecular functions, and biological processes for GO enrichment analysis (**Additional files 1**–**3**). We focused on the gene categories for which the corrected *P*-value was ≤0.05 (**Figure 4**). Ribosome-related genes were enriched in NOX as compared to CON in both the early- and the mid-exponential phase. KEGG pathway analysis (classification criteria based on KEGG pathways: http://www.genome.jp/kegg/pathway.html) was used to identify significantly changed metabolic or signal transduction pathways in NOX. The KEGG pathways controlled by differentially expressed genes at different periods of cell growth are listed in **Additional files 4**–**6**. Data analysis revealed that carbohydrate, energy, amino acid, and transcription/translation pathways were enriched in NOX vs. CON during fermentation (*Q*-values < 0.05; **Table 4**). These results were in accordance with the results reported by Vemuri et al. (2007). In the early- and mid-exponential phases, only pathways related to cell growth changed significantly, while at the late-exponential phase, numerous pathways were activated in NOX.

**Table 4:** Overview of significantly enriched KEGG-pathways in NOX.

Pathway ID	Pathway	DEGs with pathway annotation (735)	All genes with pathway annotation (3778)	*P*-value	*Q*-value^∗^
**CON harvested in the beginning of exponential phase (CBP) vs. NOX harvested in the beginning of exponential phase (NBP)**					
ko03010	Ribosome	68 (9.25%)	153 (4.05%)	5.204265e-13	4.631796e-11
ko04111	Cell cycle-yeast	58 (7.89%)	167 (4.42%)	1.470047e-06	6.541709e-05
ko04111	MAPK signaling pathway-yeast	32 (4.35%)	86 (2.28%)	7.941953e-05	2.356113e-03

**Pathway ID**	**Pathway**	**DEGs with pathway annotation (540)**	**All genes with pathway annotation (3778)**	***P*-value**	***Q*-value^∗^**

**CON harvested in the mid-exponential phase (CMP) vs. NOX harvested in the mid-exponential phase (NMP)**					
ko03008	Ribosome biogenesis in eukaryotes	31 (5.74%)	80 (2.12%)	4.245547e-08	3.820992e-06
ko04111	Cell cycle-yeast	48 (8.89%)	167 (4.42%)	5.571791e-07	2.507306e-05
ko04113	Meiosis-yeast	39 (7.22%)	163 (4.31%)	0.0005482611	1.644783e-02

**Pathway ID**	**Pathway**	**DEGs with pathway annotation c4 (1314)**	**All genes with pathway annotation (3778)**	***P*-value**	***Q*-value^∗^**

**CON harvested at the end of the exponential phase (CEP) vs. NOX harvested at the end of the exponential phase (NEP)**					
ko00640	Propanoate metabolism	43 (3.27%)	61 (1.61%)	1.092315e-08	1.081392e-06
ko00280	Valine, leucine, and isoleucine degradation	41 (3.12%)	59 (1.56%)	4.661189e-08	1.602433e-06
ko00410	Beta-Alanine metabolism	42 (3.2%)	61 (1.61%)	4.855858e-08	1.602433e-06
ko00562	Inositol phosphate metabolism	43 (3.27%)	66 (1.75%)	3.855312e-07	9.541897e-06
ko01100	Metabolic pathways	349 (26.56%)	858 (22.71%)	2.526684e-05	5.002834e-04
ko00970	Aminoacyl-tRNA biosynthesis	25 (1.9%)	41 (1.09%)	0.0005149399	8.496508e-03
ko01110	Biosynthesis of secondary metabolites	137 (10.43%)	319 (8.44%)	0.0009647584	1.322082e-02
ko00330	Arginine and proline metabolism	22 (1.67%)	36 (0.95%)	0.001068349	1.322082e-02
ko00240	Pyrimidine metabolism	42 (3.2%)	85 (2.25%)	0.00350519	3.855709e-02

*^∗^Pathways with Q-values ≤ 0.05 were significantly enriched in differentially expressed genes (DEGs).*

### Ethanol and Glycerol Metabolism are Affected at the Transcriptional Level by NADH Oxidase Overexpression

**FIGURE 5:**
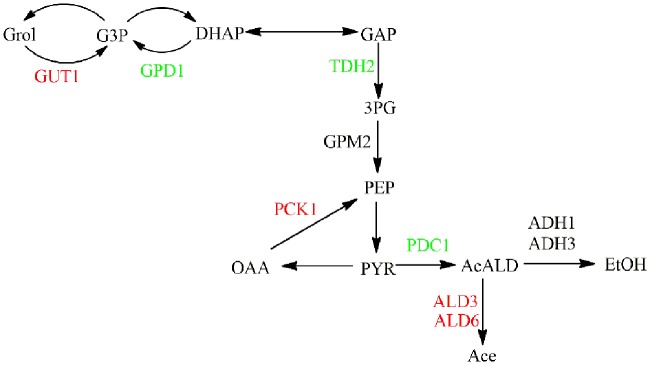
**Transcription response of strains in the late-exponential phase superimposed on ethanol and glycerol metabolism.** Genes upregulated in NOX vs. CON are indicated in red, those downregulated in NOX vs. CON are indicated in green. GUT1: glycerol kinase; GPD1: glycerol-3-phosphate dehydrogenase; TDH2: glyceraldehyde-3-phosphate dehydrogenase; GPM2: 2,3-bisphosphoglycerate-dependent phosphoglycerate mutase; PCK1: phosphoenolpyruvate carboxykinase; PDC1: pyruvate decarboxylase; ALD3: aldehyde dehydrogenase; ALD6: aldehyde dehydrogenase; ADH1: alcohol dehydrogenase; ADH3: alcohol dehydrogenase.

To further analyze the transcription response of NOX vs. CON at the end of the exponential phase with regard to ethanol and glycerol metabolism, the transcription response was superimposed on the metabolic network with the common metabolites. The glycerol assimilation pathway, involving *GUT1*, was upregulated, whereas the synthesis pathway, involving *GPD1*, was downregulated in NOX as the glycerol synthesis pathway was activated when glycolytic NADH generation was surpassed (**Figure 5**). These findings were in line with the report of Vemuri et al. (2007) and were in accordance with the results of the batch fermentation, in which the accumulation of glycerol was significantly decreased in NOX. Interestingly, in contrast to the results of Vemuri et al. (2007), the conversion of acetaldehyde to ethanol did not change significantly while the conversion of pyruvate to acetaldehyde was downregulated. The conversion of acetaldehyde to acetate was stimulated as previously reported (**Figure 5**).

According to the results of batch fermentation, the production of ethanol was improved, while in the study of Vemuri et al. (2007), it was decreased in NOX when compared with CON. These differences were reflected at the transcriptional level. In our study, the overexpression of NADH oxidase seemed to increase ethanol production mainly through the enhancement of glycolysis rather than through upregulation of ethanol synthesis genes. In terms of the ethanol production in NADH oxidase-overexpressing strains, different results have been reported. Hou et al. (2009) reported that the ethanol production rate was largely unaffected by perturbation of the cytosolic NADH. Similar to the different NADH/NAD^+^ ratios in our and other studies, the differences of ethanol production and relevant transcriptional expression could be explained in several ways as mentioned above.

### Expression of Genes Related to Thiamine Synthesis in Response to NADH Oxidase Overexpression

**FIGURE 6:**
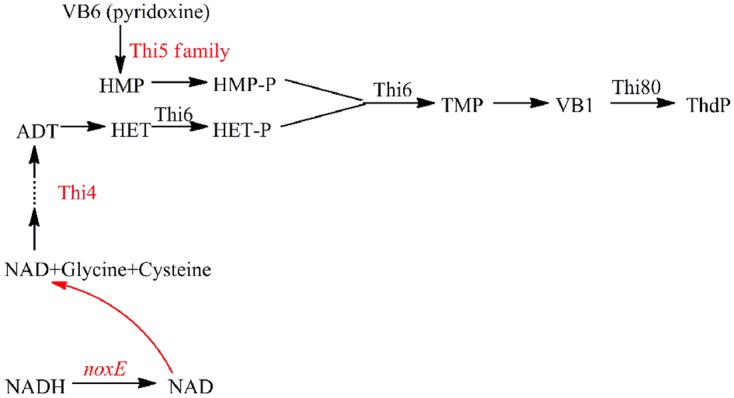
**Shematic representation of the ThdP synthesis pathway in *S. cerevisiae* showing the enzymes involved.** ADT: adenosine diphospho-5-(β-ethyl)-4-methylthiazole-2-carboxylic acid; HET: 5-Hydroxyethyl-4-methylthiazole; HET-P: 5-Hydroxyethyl-4-methylthiazole phosphate; HMP: hydroxymethylpyrimidine; HMP-P: hydroxymethylpyrimidine phosphate; TMP: thiamine phosphate; Thi4: eukaryotic thiazole biosynthetic enzyme; Thi5 family: *THI11*, *THI12*, *THI13*, and *THI5* genes; Thi6: encoding a bifunctional enzyme with both thiamine-phosphate pyrophosphorylase and HET kinase activities; Thi80: needed for both ThdP *de novo* synthesis and the conversion of external thiamine to ThdP. Genes upregulated in NOX vs. CON in the mid-exponential phase are indicated in red.

Thiamine diphosphate is an important cofactor in the metabolic pathway and the function of thiamine in stress responses has drawn much attention recently (Wolak et al., 2014). *THI* genes have a putative role in the biosynthesis of the enzyme cofactor ThdP (**Figure 6**). The four highly conserved members in *S. cerevisiae, THI11*, *THI12, THI13*, and *THI5* are known as the *THI5* gene family (Wightman and Meacock, 2003), which plays a putative role in the biosynthesis of the thiamin precursor hydroxymethylpyrimidine diphosphate. According to the transcriptome sequencing data, during fermentation, the expression of the *THI* gene family was drastically elevated in the mid-exponential phase in NOX vs. CON [*THI5*: 4.72, *THI11*: 3.67, *THI12*: 2.97, and *THI13*: 2.95 (values are log_2_ ratios)]. Wightman and Meacock (2003) described that in the *Saccharomyces sensu stricto* group, yeasts have multiple copies of *THI5*, and a great many of the amplified genes in *THI5*-overexpressing strains found near the telomeres, including those in duplicated *THI5* blocks, encode proteins involved in sugar uptake and metabolism (e.g., *HXT*, *SUC*, *MAL*, etc.). These amplifications of sugar uptake genes probably confer rapid uptake mechanisms leading to a greater glycolytic flux to pyruvate. In accordance herewith, in our study, the expression of hexose transport genes *HXT6* and *HXT16* was significantly upregulated (6.97 and 3.01, respectively) in NOX at the mid-exponential phase. In batch fermentation, the glucose uptake of NOX was much faster than that of CON, and thiamine accelerated the cell growth in NOX, especially in the mid-exponential phase (Wolak et al., 2014). At the end of the exponential phase, the expression of the *THI5* gene family did not significantly change in NOX. This could be explained by the fact that after 29.5 h of fermentation, the glucose in the NOX medium was exhausted and the fermentation completed; thus, less ThdP was needed as compared to the mid-exponential phase.

THI4 is a eukaryotic thiazole biosynthetic enzyme that catalyzes the conversion of ADT to HET, which was shown to protect cells against destabilizing conditions (Wolak et al., 2014). In our study, overexpression of the NADH oxidase regulated the intracellular redox balance by increasing the demand and oxidation of NADH as well as the regeneration of NAD^+^, likely improving the synthesis of thiazole (*THI4: 4.27*). However, unlike the *THI5* gene family and the *THI4* gene, the expression of downstream genes ThdP synthesis genes *THI6* (encoding a bifunctional enzyme with both thiamine-phosphate pyrophosphorylase and HET kinase activities) and *THI80* (needed for both ThdP *de novo* synthesis and the conversion of external thiamine to ThdP; Mojzita and Hohmann, 2006) was not significantly changed, even at the mid-exponential phase. Similarly, the expression of genes encoding ThdP-dependent enzymes pyruvate decarboxylase and pyruvate dehydrogenase was not significantly changed. The positive regulator of ThdP, THI2 (Mojzita and Hohmann, 2006), was upregulated only at the end of the exponential phase. These results indicated that overexpression of the NADH oxidase could stimulate thiamine, involved in redox balance maintenance in yeast cells, partly independently of the functions of ThdP and ThdP-dependent enzymes (Wolak et al., 2014).

### Transcriptional Response of the Global Transcription Factor HAP4 to the NADH Oxidase Overexpression

At the end of the exponential phase, the expression of *HAP4* was remarkably increased (4.06) upon NADH oxidase overexpression. Previous studies found that *HAP4* overexpression provokes changes that also occur during the diauxic shift—a type of starvation response (Blom et al., 2000; Lascaris et al., 2002). In our study, after 29.5 h of fermentation, residual glucose was almost undetectable in NOX culture and the cells had started to use the ethanol as indicated by the lowered concentration of ethanol at this time point, suggestive of a diauxic shift. Schuurmans et al. (2008) reported that overexpression of HAP4 in glucose-rich growth condition results in increased mitochondrial biogenesis. In addition, genes involved in oxidative phosphorylation were strongly upregulated. According to our transcriptome data, various genes involved in oxidative phosphorylation, such as *YMR145C* (encoding NADH dehydrogenase); *COR1*, *QCR6*, *QCR7*, and *QCR8* in Complex III; and *COX6A* and *COX15* (*YER141W*) in Complex IV, were significantly upregulated in NOX at the end of the exponential phase. Overexpression of HAP4 negatively affects the transcription of a small set of genes involved in zinc metabolism mediated through decreased expression of the transcription factor Zap1 (YJL056C; Lascaris et al., 2002). The motif bound by the Zap1 regulator, which controls zinc transport and homeostasis, was strongly downregulated, suggesting that there is a much lower demand for Zn in strains overexpressing HAP4 (Schuurmans et al., 2008). Our transcriptome data revealed that the expression of several genes involved in zinc metabolism [*YGL258W* (-1.04), *SNO1* (-1.59), *RTC4* (- 1.33) etc.] was reduced. The changes were much smaller than those reported by Schuurmans et al. (2008), who found that overexpression of HAP4 increased the expression of a high-affinity glucose transporter and significantly induced genes associated with growth on non-fermentable carbon sources (e.g., *ADH2* and *FBP1*). In our study, the high-affinity hexose transporter *HXT6* (7.88) was significantly upregulated in NOX at the late-exponential phase. In accordance with a diauxic shift, the expression of *FBP1* (2.12) was increased.

### Overexpression of the NADH Oxidase Regulates Apoptosis Related Genes

Changes in redox homeostasis through the fermentation process would increase the accumulation of ROS (Ayer et al., 2014). Xu et al. (2010) found that overexpression of the water-forming NADH oxidase in *T. glabrata* decreased the ROS production, protecting the yeast cells from ROS damage. ROS are widely recognized as crucial cell death regulators and have been connected to many of the known apoptotic pathways in yeast. Carmona-Gutierrez et al. (2010) have comprehensively analyzed apoptosis in yeast. Since the discovery of yeast apoptosis, multiple yeast orthologs of crucial mammalian apoptotic proteins have been identified. Yeast bears at least one ortholog of mammalian caspases: the metacaspase Yca1. Numerous cell death scenarios have been shown to depend on Yca1. Apoptotic death mediated by the apoptosis-inducing factor Nuc1, the yeast homolog of endonuclease-G, does not require Yca1.

**FIGURE 7:**
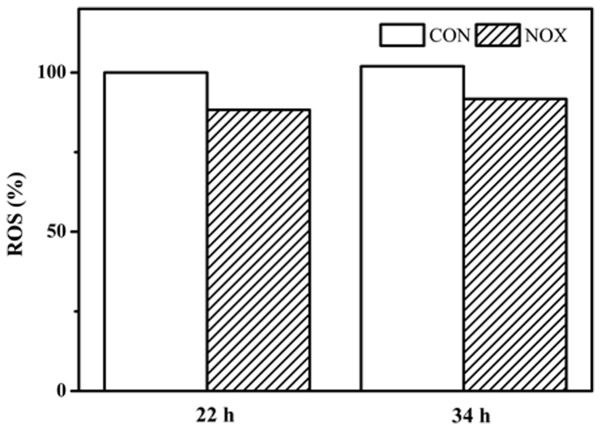
**Reactive oxygen species (ROS) prodution in CON and NOX.** In the batch fermentation, ROS production was measured at 22 and 34 h for CON and NOX. Each value is an average of three parallel replicates. ROS production was expressed as a ratio relative to that in CON cells collected at 22 h.

To determine whether the overexpression of *noxE* in *S. cerevisiae* could decrease ROS, ROS production by CON and NOX cells was evaluated (**Figure 7**). In both CON and NOX strains, ROS production was not significantly increased at 34 h as compared to 22 h. The production of ROS was approximately 10% lower in NOX than in CON at both time points. This result was consistent with the results of Xu et al. (2010).

To analyze the effect of NADH oxidase overexpression on yeast apoptosis, we checked the expression levels of apoptosis-related factors and proteins reported by Carmona-Gutierrez et al. (2010). We found that *Nuc1* was downregulated at the mid-exponential phase (-1.03) and at the end of exponential phase (-3.57). In addition, karyopherin Kap123, which is necessary for Nuc1-mediated apoptosis (Carmona-Gutierrez et al., 2010), was downregulated (-1.29) at the end of the exponential phase. Similar to other apoptotic players, Nuc1 exerts lethal and vital functions as illustrated by deletion of the gene, which inhibits apoptosis when mitochondrial respiration is enhanced but increases necrotic death when oxidative phosphorylation is repressed (Ayer et al., 2014). As indicated by the HAP4 expression data mentioned above, the NOX cells might undergo a diauxic shift, which would enhance mitochondrial respiration, and in this condition, the downregulated Nuc1 might inhibit apoptosis. Büttner et al. (2007) also reported that at the beginning of the stationary phase, there is a Nuc1-dependent apoptotic clearance. The higher cell density of NOX compared to CON (**Figure 1B**) at the beginning of the stationary phase in our study supported this viewpoint.

In addition to ROS, the small signaling molecules NO and ammonia regulate yeast apoptosis. During the development of multicellular yeast colonies, ammonia accumulates in the center of the colonies and triggers the death of older cells, allowing young cells on the rim to exploit the released nutrients. Consistently, lack of the transcription factor Sok2, which results in the inability to produce ammonia, leads to diffuse death throughout the population and diminishes the life span of the colony (Carmona-Gutierrez et al., 2010). In the current study, the transcription factor Sok2 was upregulated at the end of the exponential phase (2.39).

In conclusion, overexpression of the NADH oxidase in *S. cerevisiae* regulated several apoptosis related genes. However, apoptosis was not induced on a large scale in our study, as evidenced by both cell growth and transcription; therefore, the detailed relationship between overexpression of NADH oxidase and yeast apoptosis could not be determined in this study and requires further research.

### Heterologous Expression of NADH Oxidase Enhances *S. cerevisiae* Growth under Hyperosmotic Stress

It has been reported that the osmotolerance of *T. glabrata* cells can be enhanced by the introduction of water-forming NADH oxidase (Xu et al., 2010). To analyze the effect of the enzyme on osmotic stress in *S. cerevisiae*, we measured the osmolarity of the media of NOX and CON after 30 h of fermentation. The osmolarity of the fermentation medium of CON was 1834 ± 6 mOsmol/kg, while that of NOX was 2028 ± 2 mOsmol/kg, which was a 10.58% increase as compared to CON. Since NOX produced more ethanol than CON (**Figure 1A**), it is conceivable that the NOX strain was under higher osmotic stress.

**FIGURE 8:**
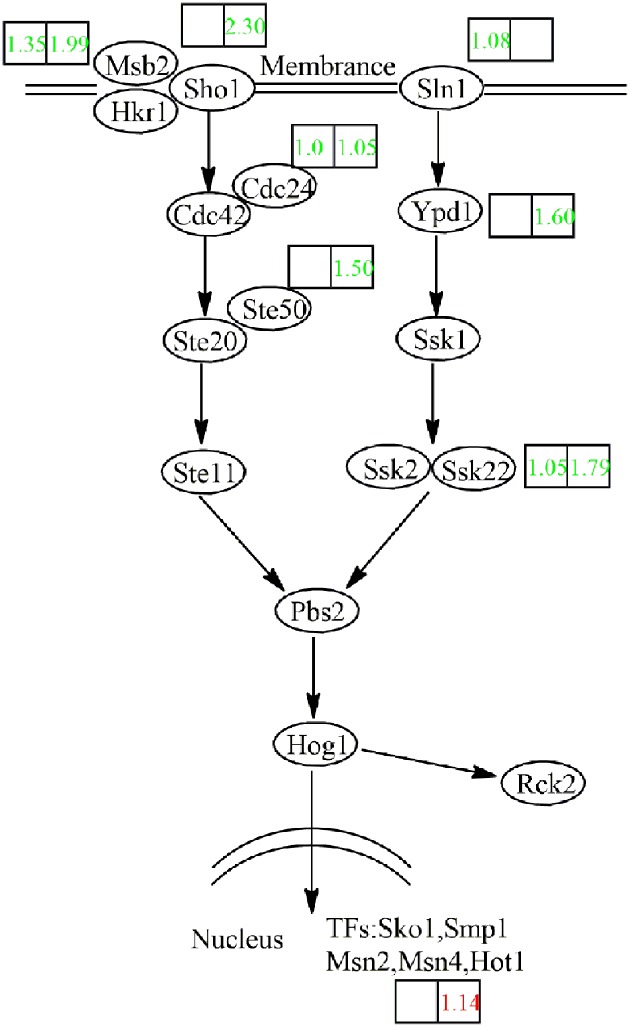
**Transcriptional response of the HOG pathway in NOX to overexpression of the NADH oxidase.** Log_2_ expression ratios are presented in boxes (left box: CMP vs. NMP; right box: CEP vs. NEP). Genes upregulated in NOX vs. CON are indicated in red, those downregulated in NOX vs. CON are indicated in green. Msb2: transmembrane transporter; Hkr1: transmembrane transporter; Sho1: osmosensor; Cdc42: cell division control protein; Cdc24: cell division control protein; Ste50: bZIP factor; Ste20: mitogen-activated protein kinase kinase kinase kinase; Ste11: mitogen-activated protein kinase kinase kinase; Sln1: sensor histidine kinase; Ypd1: phosphorelay intermediate protein; Ssk1: response regulator; Ssk2: mitogen-activated protein kinase kinase kinase; Ssk22: mitogen-activated protein kinase kinase kinase; Pbs2: mitogen-activated protein kinase kinase; Hog1: mitogen-activated protein kinase; Rck2: serine/threonine protein kinase; Sko1: cAMP response element-binding protein; Smp1: MADS-box transcription factor; Msn2: zinc finger protein; Msn4: zinc finger protein; Hot1: transcription factor.

The HOG MAPK pathway plays a role in the adaptation of yeast cells to high osmotic pressure (O’Rourke et al., 2002). Under continued activation of the HOG pathway in case of chronic osmotic stress, the cells die (Jiang et al., 2004). In **Figure 8**, the HOG pathway is superimposed on our transcriptome data. During the fermentation of NOX, the HOG pathway seemed not to be activated even at the end of the logarithmic phase, although the osmotic stress was truly higher than in CON. The low production of glycerol in NOX confirmed inactivity of the HOG pathway.

**FIGURE 9:**
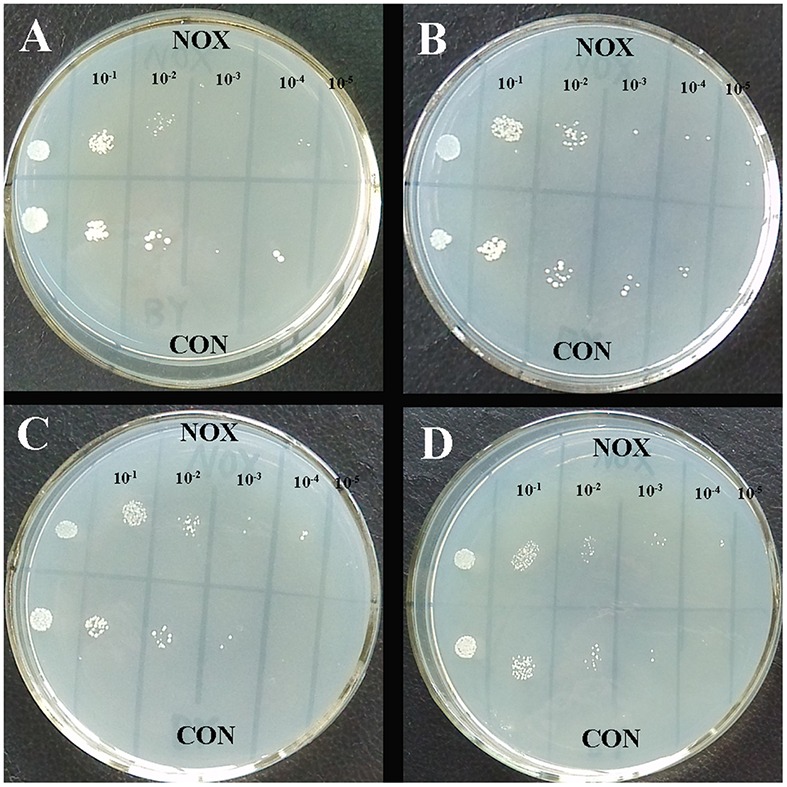
**Resistance of CON and NOX to osmotic stress. (A)** 0 g/L NaCl; **(B)** 9 g/L NaCl; **(C)** 27 g/L NaCl; **(D)** 45 g/L NaCl.

To further study the survival ability of NOX and CON under hyperosmotic stress, qualitative sensitivity at different concentrations of NaCl was measured as colony-forming units at different dilution levels (**Figure 9**; Xu et al., 2010). Organism suspensions were OD-standardized before sample application (2 μL). After 48 h, similar colonies of NOX and CON were observed at initial concentration in four plates (leftmost column in **Figures 9A–D**). However, the engineered NOX strain had a 10-fold higher survival rate than CON at 0, 9, and 27 g/L of NaCl (**Figures 9A–C**). Larger and more colonies of NOX were observed at 10^-4^ dilution and 45 g/L NaCl as compared to CON. Similar results were reported by Xu et al. (2010) in *T. glabrata*. Our results suggested that heterologous expression of NADH oxidase in *S. cerevisiae* confers protection to *S. cerevisiae* under hyperosmotic conditions, which will reduce the cells death.

### Confirmation of Transcriptome Profiling Results by qRT-PCR

**FIGURE 10:**
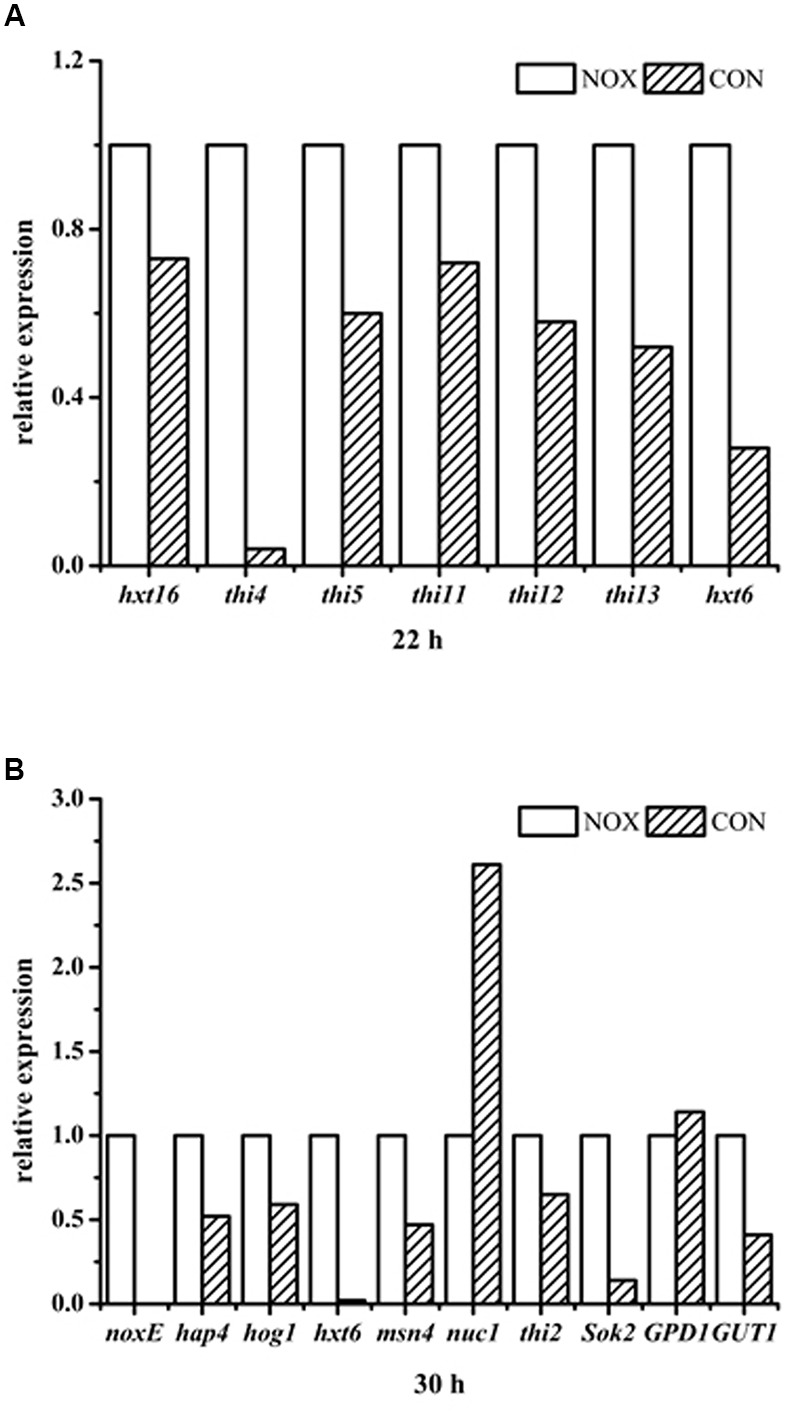
**qRT-PCR results. (A)** relative expression of genes in NOX vs. CON after 22 h of fermentation; **(B)** relative expression of genes in NOX vs. CON after 30 h of fermentation.

The levels of transcription of various key genes mentioned above were verified by qRT-PCR (**Figure 10**). As shown in **Figure 10B**, *noxE* was not expressed in the control strain CON, and it was effectively expressed in the recombinant strain NOX. The expression levels of *THI4* and *HXT6* were significantly upregulated in NOX at 22 h (**Figure 10A**). The difference in *HXT6* expression in NOX vs. CON was more pronounced at 30 h than at 22 h. The expression of *GUT1*, involved in the glycerol assimilation pathway, was upregulated, whereas *GPD1*, involved in the glycerol synthesis pathway, was not differentially regulated. With respect to HOG pathway, the expression levels of *Hog1* and *msn4* (a transcription factor) were not significantly changed, in accordance with the RNA-seq results. With regard to yeast apoptosis, the expression of *Nuc1* was downregulated and the expression of *Sok2* was upregulated in the NADH oxidase-overexpressing strain. Detailed results and calculations are presented in **Additional file 7**. The results of qRT-PCR indicated that the RNA-seq results are accurate and reliable, strengthening our conclusions from these data.

## Conclusion

This study unraveled the response *S. cerevisiae* to overexpression of a water-forming NADH oxidase at the metabolic and the transcriptional level. In batch fermentation, we observed reduced glycerol and increased glucose consumption, ethanol, and cell growth in the NOX strain, showing the potential usability of the recombinant *S. cerevisiae* strain in large-scale ethanol production. In addition, overexpression of the NADH oxidase conferred protection to *S. cerevisiae* under hyperosmotic conditions. The expression of multiple genes was changed at the three time points evaluated. Compared with CON, various genes related to thiamine synthesis were upregulated during NOX fermentation. After 29.5 h of fermentation, the glucose in NOX culture was almost exhausted and the strain underwent a diauxic shift, with significant upregulation of the global transcriptional factor HAP4. The heterologous expression of NADH oxidase in *S. cerevisiae* lowered the ROS production. The yeast apoptosis-inducing factor Nuc1 was downregulated and transcription factor Sok2 was upregulated, collectively suggestive of the inhibition of apoptosis in the NOX strain. Thiamine plays an important role in the maintenance of redox balance, which is essential to sustain metabolism and growth. We reason that the upregulation of HAP4 resulted in enhanced mitochondrial respiration, which is essential for the downregulation of Nuc1 to inhibit apoptosis. In addition, because of the improved osmotolerance, the HOG pathway, which might be connected to Nuc1-induced apoptosis, was not activated. Together, these transcriptional changes in response to stresses under the overexpression of NADH oxidase improved the metabolism and growth of *S. cerevisiae* cells under the given conditions. This study put forward possible relationships between the water-forming NADH oxidase and thiamine synthesis, the global transcriptional factor HAP4, apoptosis, and osmotolerance, providing several new directions for further study of the NADH oxidase and the molecular mechanism underlying the transcriptional changes.

## Author Contributions

XS participated in the design of the study, constructed the plasmids and strains, participated in the experiments, analyzed the RNA-seq data, drafted the manuscript, and revised the manuscript. YZ participated in the fermentation experiments. YC participated in the design of the study. CZ helped to analyze the data and revise the manuscript. HY conceived of the study, and participated in its design. All authors have read and approved the final manuscript.

## Conflict of Interest Statement

The authors declare that the research was conducted in the absence of any commercial or financial relationships that could be construed as a potential conflict of interest.

## Supplementary material

ADDITIONAL FILE S1 **Gene ontology (GO) enrichment analysis for CBP and NBP cells**.

ADDITIONAL FILE S2 **Gene ontology enrichment analysis for CMP and NMP cells**.

ADDITIONAL FILE S3 **Gene ontology enrichment analysis for CEP and NEP cells**.

ADDITIONAL FILE S4 **KEGG pathway analysis for CBP and NBP cells**.

ADDITIONAL FILE S5 **KEGG pathway analysis for CMP and NMP cells**.

ADDITIONAL FILE S6 **KEGG pathway analysis for CEP and NEP cells**.

ADDITIONAL FILE S7 **Results of qRT-PCR of *S. cerevisiae***.

## Abbreviations

ADT: adenosine diphospho-5-(β-ethyl)-4-methylthiazole-2-carboxylic acid
CON: *Saccharomyces cerevisiae* BY4741 containing the empty plasmid pYX212
HET: 5-hydroxyethyl-4-methylthiazole
NOX: *S. cerevisiae* BY4741 overexpressing the water-forming NADH oxidase encoded by *noxE*
ROS: reactive oxygen species
ThdP: thiamine diphosphate
